# OP43 Incorporating Mathematical Modeling To Improve Accuracy Of Budget Impact Analysis: Using Screening For Hepatitis C As An Example

**DOI:** 10.1017/S0266462324001053

**Published:** 2025-01-07

**Authors:** William Wong

## Abstract

**Introduction:**

The majority of those infected with chronic hepatitis C (CHC) are asymptomatic. Population screening has proven to be both effective and cost effective. When considering whether to implement screening or not, the uncertainty of the budget impact plays an important role. This study aims to develop methods that improve the accuracy of budget impact analysis for a one-time CHC screening program.

**Methods:**

We developed a back-calculation mathematical model that employs a Markov chain Monte Carlo algorithm to estimate the prevalence and proportion of undiagnosed CHC. Subsequently, we utilized a state-transition model to assess the budget impact of two strategies: (i) no screening; and (ii) screen-and-treat with direct-acting antiviral (DAA) for individuals born between 1945 and 1965 (“baby-boomer” birth cohort). Model data were gathered from published literature. Our analysis adopted a Canadian provincial payer perspective, employed a 10-year time horizon, and followed best-practice recommendations by not applying discounting.

**Results:**

For individuals born between 1945 and 1965, the estimated prevalence of CHC was 1.74 percent (95% confidence interval [CI]: 1.52, 2.30) with an undiagnosed proportion of 15.72 percent (95% CI: 11.27, 18.54). The initial budget impact analysis indicated an additional cost of CAD61.5 million (USD45.0 million) over 10 years for screening related individuals for CHC in Ontario. With these updated prevalence and undiagnosed proportion estimates, our projection suggests a 29.6 percent reduction in the budget impact, now estimated at CAD43.3 million (USD31.7 million).

**Conclusions:**

By comparing the budget impact of the CHC screening strategy with other recommended health services and technologies in Ontario, we have concluded that CHC screening may be considered affordable. To enhance the accuracy of budget impact analysis for population-level screening decision-making, it is crucial to develop precise methodologies for estimating the underlying prevalence and undiagnosed proportions.

